# Supplementary material for: Development and primary validation of the School Health Assessment Tool for Primary Schools (SHAT-PS)
Source: PeerJ. 2021 Dec 13;9:e12610. doi: 10.7717/peerj.12610 (PMC8675247; doi:10.7717/peerj.12610)
Supplement: Supplemental Information 1 [file peerj-09-12610-s001.docx]

**پرسشنامه سنجش سلامت مدرسه - مقطع ابتدایی**

| **عوامل سلامت مدرسه** | **سوالات** | **خیر اصلا** | **کمی** | **زیاد** | **خیلی زیاد** |
| --- | --- | --- | --- | --- | --- |
| **سیاست های سلامت مدرسه** | 1- مدرسه برای حقوق و وظایف افراد قوانین مشخصی دارد. |  |  |  |  |
|  | 2- تشکل سازمان یافته سلامت در مدرسه وجود دارد (مثل بهداشت یاران، پیشگامان سلامت، و مروجین سلامت). |  |  |  |  |
|  | 3- امکانات آموزشی مدرسه کافی است. |  |  |  |  |
|  | 4- امکانات رفاهی مدرسه کافی است. |  |  |  |  |
|  | 5- مدرسه امکانات لازم را در صورت بروز حوادث غیر مترقبه (مثل آتش­سوزی، زلزله و غیره) دارد. |  |  |  |  |
|  | 6- مدرسه برنامه های دائمی و منظمی برای فعالیت های تفریحی و آموزشی خارج از مدرسه برای دانش آموزان دارد. |  |  |  |  |
|  | 7- رضایت شغلی کارکنان مدرسه برآورده می شود. |  |  |  |  |
| **پیوند اجتماعی** | 8- خانواده­ها در خصوص ارتقاء سلامت دانش­آموزان با مدرسه همکاری می­کنند. |  |  |  |  |
|  | 9- موسسات حمایتی و خیریه با مدرسه در جهت ارتقاء سلامت مدرسه همکاری می­کنند. |  |  |  |  |
|  | 10- رسانه ها و صدا سیما در جهت ارتقاء سلامت در مدارس برنامه های آموزشی تهیه و پخش می کنند. |  |  |  |  |
|  | 11- وزارت و اداره آموزش و پرورش بودجه کافی به  مدارس اختصاص می دهد. |  |  |  |  |
| **آموزش سلامت** | 12- مدرسه برنامه­های آموزشی مرتبط با سلامت برای دانش آموزان برگزار می­کند. |  |  |  |  |
|  | 13- پوسترها، استندها و بوردهای آموزش سلامت و بهداشت در کلاس ها، راهروها و سالن ها نصب شده است. |  |  |  |  |
|  | 14- کمک­های اولیه اورژانسی و امداد به دانش­آموزان آموزش داده می­شود. |  |  |  |  |
| **ورزش** | 15- حداقل یک ورزش به طور تخصصی در مدرسه آموزش داده می شود، مثل والیبال، بسکتبال، هندبال، فوتبال و غیره. |  |  |  |  |
|  | 16- مسابقات ورزشی در مدرسه برگزار می شود. |  |  |  |  |
|  | 17- ساعات کافی در هفته به ورزش اختصاص داده می شود. |  |  |  |  |
|  | 18- مدرسه امکانات ورزشی کافی دارد. |  |  |  |  |
| **خدمات سلامت** | 19- مدرسه مربی بهداشت با تجربه دارد. |  |  |  |  |
|  | 20- وضعیت سلامت دانش آموزان ارزیابی و در پرونده سلامت آنها ثبت می شود. |  |  |  |  |
|  | 21- جعبه کمک­های اولیه به اندازه کافی برای دانش­آموزان و پرسنل مدرسه موجود است. |  |  |  |  |
|  | 22- مربیان بهداشت خدمات سلامتی اورژانسی به دانش­آموزان و کارکنان مدرسه ارائه می­دهند. |  |  |  |  |
|  | 23- معلمان مشکلات سلامتی دانش­آموزان را با والدین در میان می­گذارند. |  |  |  |  |
|  | 24- وضعیت واکسیناسیون دانش آموزان چک می شود. |  |  |  |  |
| **تغذیه** | 25- غذاهای مدرسه با رعایت اصول بهداشتی تهیه می­شوند. |  |  |  |  |
|  | 26- در مدرسه غذاهای مفید و مغذی ارایه می­شود. |  |  |  |  |
|  | 27- اطلاعات مکفی در مورد تغذیه سالم به دانش آموزان ارائه می شود. |  |  |  |  |
|  | 28- آب آشامیدنی بهداشتی و مطمئن در دسترس است. |  |  |  |  |
|  | 29- بر بهداشت بوفه نظارت می شود. |  |  |  |  |
| **خدمات روانشناسی** | 30- دانش­آموزان ترغیب می­شوند که در یادگیری دروس فعال باشند. |  |  |  |  |
|  | 31- از بروز قلدری و خشونت بین دانش­آموزان ممانعت می­شود. |  |  |  |  |
|  | 32- دانش آموزان دارای مشکلات خاص (رفتاری و یادگیری) شناسایی و به واحد مربوطه ارجاع داده می شوند. |  |  |  |  |
|  | 33- آموزش­های روانشناختی لازم به کارکنان مدرسه و والدین ارایه می شود. |  |  |  |  |
|  | 34- دانش آموزان از حضور در مدرسه لذت می برند. |  |  |  |  |
| **محیط فیزیکی** | 35- سرویس­های بهداشتی تمیز هستند. |  |  |  |  |
|  | 36- تعداد سرویس های بهداشتی کافی است. |  |  |  |  |
|  | 37- آبخوری ها تمیز و بهداشتی هستند. |  |  |  |  |
|  | 38- تعداد آبخوری ها کافی است. |  |  |  |  |
|  | 39- نور کلاس ها کافی است. |  |  |  |  |
|  | 40- سیستم گرمایشی در کلاس­ها مناسب و کافی است. |  |  |  |  |
|  | 41- سیستم سرمایشی در کلاس­ها مناسب و کافی است. |  |  |  |  |
|  | 42- مدرسه به طور منظم از نظر امنیت ساختمان­ها، پنجره­ها و تجهیزات بررسی می­شود. |  |  |  |  |
|  | 43- میز و صندلی های کلاس ها استاندارد و راحت هستند. |  |  |  |  |
|  | 44- مساحت مدرسه با تعداد دانش آموزان تناسب دارد. |  |  |  |  |
|  | 45- مساحت کلاس ها با تعداد دانش آموزان تناسب دارد. |  |  |  |  |
|  | 46- محیط مدرسه شاد است. |  |  |  |  |
|  | 47- در مدرسه فضای سبز وجود دارد. |  |  |  |  |

روش نمره گذاری به صورت زیر می باشد:

خیر اصلا: 1 امتیاز، کمی: 2 امتیاز، زیاد: 3 امتیاز، خیلی زیاد: 4 امتیاز

همه سوالات به صورت مستقیم نمره گذاری می شوند و هیچ یک از سوالات دارای نمره گذاری معکوس نمی باشد.

این پرسشنامه باید توسط مدیر، معلم، یا مربی بهداشت مدرسه پاسخ داده شود.
