# Supplementary material for: Development and primary validation of the School Health Assessment Tool for Primary Schools (SHAT-PS)
Source: PeerJ. 2021 Dec 13;9:e12610. doi: 10.7717/peerj.12610 (PMC8675247; doi:10.7717/peerj.12610)
Supplement: Supplemental Information 2 [file peerj-09-12610-s002.docx]

**Appendix**

**School Health Assessment Tool for Primary Schools (76-item version)^[[1]](#footnote-1)^**

| **Factors** | **Items** | **Not at all** | **A little** | **Quite a lot** | **Very much** |
| --- | --- | --- | --- | --- | --- |
| School health policies | 1- Health educational posters, stands, and boards have been installed in classrooms, hallways, and halls. |  |  |  |  |
|  | 2- School has specific rules for the rights and duties of individuals. |  |  |  |  |
|  | 3- Staff smoking on the school’s territory is prohibited. |  |  |  |  |
|  | 4- Students are involved in some school decisions. |  |  |  |  |
|  | 5- There is a distinctive health organization at school (such as health care providers, health pioneers, and health promoters). |  |  |  |  |
|  | 6- School's educational facilities are sufficient. |  |  |  |  |
|  | 7- School's amenities are sufficient. |  |  |  |  |
|  | 8- School has regular programs for out-of-school recreational and educational activities for students. |  |  |  |  |
| Community connections | 9- Families work with the school to improve students’ health. |  |  |  |  |
|  | 10- Supportive and charitable organizations work with the school to promote school health. |  |  |  |  |
|  | 11- Media and TV provide educational programs to promote health at schools. |  |  |  |  |
|  | 12- The Ministry and the Department of Education allocate sufficient budget and funds to the school. |  |  |  |  |
| Health education | 13- Health-related routine behaviors are taught to students. |  |  |  |  |
|  | 14- Students work together to keep the school clean and tidy. |  |  |  |  |
|  | 15- School organizes health-related educational workshops, conventions, and programs for students. |  |  |  |  |
|  | 16- School organizes health-related educational workshops, conventions, and programs for parents. |  |  |  |  |
|  | 17- School organizes health-related educational workshops, conventions, and programs for school staff. |  |  |  |  |
|  | 18- First aid training is provided to students. |  |  |  |  |
|  | 19- Adequate hours during the week are devoted to health education. |  |  |  |  |
|  | 20- There is sufficient course or syllabus for health education. |  |  |  |  |
| Physical activity and education | 21- There is a skilled physical education teacher at school. |  |  |  |  |
|  | 22- At least one sport is taught professionally at school, such as volleyball, basketball, handball, football, etc. |  |  |  |  |
|  | 23- Sport competitions are held at school. |  |  |  |  |
|  | 24- Adequate hours of the week are devoted to sport at school for students. |  |  |  |  |
|  | 25- Adequate hours of the week are devoted to playing at school for students. |  |  |  |  |
|  | 26- School has enough sport facilities. |  |  |  |  |
| Health services | 27- Students' health status is assessed and registered in their health records. |  |  |  |  |
|  | 28- There are enough first aid kits for students and school staff. |  |  |  |  |
|  | 29- There is a skilled nurse practitioner/physician at school. |  |  |  |  |
|  | 30- School nurse(s) provide emergency health care services to students and school staff. |  |  |  |  |
|  | 31- Teachers share students' health/educational issues with their parents. |  |  |  |  |
|  | 32- School has an equipped place to provide health services. |  |  |  |  |
|  | 33- There is sufficient cleaning staff at school. |  |  |  |  |
|  | 34- The vaccination status of students is checked. |  |  |  |  |
| Nutrition | 35- School meals are prepared according to hygienic principles. |  |  |  |  |
|  | 36- School offers healthy and nutritious foods. |  |  |  |  |
|  | 37- Healthy and safe drinking water is available. |  |  |  |  |
|  | 38- School has buffet or restaurant with enough space to sit. |  |  |  |  |
|  | 39- Buffet’s hygiene is monitored. |  |  |  |  |
|  | 40- Foods and snacks are served according to students' tastes. |  |  |  |  |
|  | 41- Adequate information about healthy eating is provided to students. |  |  |  |  |
| Psychological services and counselling | 42- There is a skilled psychologist/counselor at school. |  |  |  |  |
|  | 43- Students are encouraged to be active in learning the lessons. |  |  |  |  |
|  | 44- Bullying and violence among students are prevented. |  |  |  |  |
|  | 45- Psychological counseling and support services are provided to students with social, emotional, and physical problems. |  |  |  |  |
|  | 46- School submits mental health reports of students in their records. |  |  |  |  |
|  | 47- Necessary psychological training is provided to teachers and school staff. |  |  |  |  |
|  | 48- Necessary psychological training is provided to parents. |  |  |  |  |
|  | 49- How to communicate and interact healthily and effectively is taught to students. |  |  |  |  |
|  | 50- Students with special problems (behavioral and learning) are identified and referred to the relevant specialists. |  |  |  |  |
|  | 51- Students enjoy attending school. |  |  |  |  |
| Physical environment | 52- Restrooms are clean. |  |  |  |  |
|  | 53- The number of restrooms is sufficient. |  |  |  |  |
|  | 54- Drinking fountains or water coolers are clean. |  |  |  |  |
|  | 55- The number of drinking fountains is sufficient. |  |  |  |  |
|  | 56- The number of ventilators in different parts of the school is sufficient. |  |  |  |  |
|  | 57- Classroom lighting is sufficient. |  |  |  |  |
|  | 58- The heating system in classrooms is appropriate and sufficient. |  |  |  |  |
|  | 59- The cooling system in classrooms is appropriate and sufficient. |  |  |  |  |
|  | 60- Classroom windows are double glazed. |  |  |  |  |
|  | 61- Classroom desks and chairs are standard and comfortable. |  |  |  |  |
|  | 62- The area of the school is proportional to the number of students. |  |  |  |  |
|  | 63- The area of the classrooms is proportional to the number of students. |  |  |  |  |
|  | 64- School has an appropriate manner for sanitary waste disposal. |  |  |  |  |
|  | 65- School’s physical environment is happy (colors, decorations, layouts, etc.) |  |  |  |  |
|  | 66- There is sufficient green space at school. |  |  |  |  |
|  | 67- School has a well-equipped library. |  |  |  |  |
|  | 68- School has a well-equipped laboratory. |  |  |  |  |
| School staff’s health | 69- Psychological services are provided for school staff. |  |  |  |  |
|  | 70- Sport facilities are provided for school staff. |  |  |  |  |
|  | 71- School staff's job satisfaction is met. |  |  |  |  |
|  | 72- Teachers receive adequate salaries. |  |  |  |  |
|  | 73- Teachers have experienced assistants for teaching. |  |  |  |  |
| Security and safety | 74- There are hidden cameras in public areas inside and outside of the school. |  |  |  |  |
|  | 75- School has necessary facilities in case of unexpected events (such as fire, earthquake, etc.). |  |  |  |  |
|  | 76- School is regularly inspected for the safety of buildings, windows, and equipment. |  |  |  |  |

1. For use, translation or application of any version of the School Health Assessment Tool for Primary Schools (SHAT-PS) please contact Maryam Kazemitabar ([maryam.kazemi64@ut.ac.ir](mailto:maryam.kazemi64@ut.ac.ir)) and Danilo Garcia ([danilo.garcia@icloud.com](mailto:danilo.garcia@icloud.com)). [↑](#footnote-ref-1)
